# Experience of Sustained Low-Efficiency Dialysis (SLED) in an Intensive Care Unit of a Quaternary Care Hospital

**DOI:** 10.7759/cureus.54376

**Published:** 2024-02-17

**Authors:** Saleem Sharieff, Wajid Rafai, Adil Manzoor, Asim Idrees, Burhan Ahmad, Madiha Ghulam, Muhammad Usman Shabbir

**Affiliations:** 1 Critical Care Medicine, Pakistan Kidney and Liver Institute and Research Centre, Lahore, PAK; 2 Critical Care Medicine, Grand River Hospital, Kitchener, CAN; 3 Nephrology, Pakistan Kidney and Liver Institute and Research Centre, Lahore, PAK; 4 Medicine, Pakistan Kidney and Liver Institute and Research Centre, Lahore, PAK

**Keywords:** prognostic marker, shock, hemodynamically unstable, dialysis, renal replacement therapy

## Abstract

Background: In critically ill patients, sustained low-efficiency dialysis (SLED) has become a viable option for treating acute kidney injury (AKI) instead of continuous renal replacement therapy (CRRT). This study aimed to evaluate clinical outcomes in critically ill patients receiving SLED.

Material and methods: In our ICU, we performed a retrospective cohort study on hemodynamically unstable patients requiring dialysis in the form of SLED. Demographics, clinical, and biochemical variables were analyzed.

Results: A total of 58 patients were enrolled in the study. The mean age was 48.58 ± 15 with a male-to-female ratio of 3:1. Higher APACHE II score, high international normalized ratio, thrombocytopenia, and septic shock were found to be poor prognostic markers, with an overall observed mortality of 56.9%.

Conclusion: SLED can be considered as an alternative to CCRT for selected hemodynamically unstable patients requiring renal replacement therapy.

## Introduction

Acute kidney injury (AKI) is not uncommon in intensive care units (ICUs) requiring intermittent hemodialysis (IHD) or continuous renal replacement therapy (CRRT) depending on the hemodynamic status of the patient. Hypotension is a common complication during IHD, causing inadequate fluid and solute removal [1]. Although CRRT is more tolerable in unstable patients, it is a complex and expensive modality requiring continuous monitoring, anticoagulation [2], patient immobility, other support lines, and equipment. All this causes a longer ICU to stay and an increased financial burden.

Sustained low-efficiency dialysis (SLED) is a hybrid form of renal replacement therapy between conventional intermittent hemodialysis (IHD) and continuous renal replacement therapy (CRRT) [3,4]. Advantages of SLED are efficient clearance of small solutes, good hemodynamic tolerability, flexible treatment schedules, and reduced cost. As opposed to IHD, in SLED, the blood and dialysate flow rates are slower while the duration of the session is longer; this leads to hemodynamic tolerance and efficient clearance of solutes, with less workload for ICU staff as compared to CCRT [3-7].

Since CRRT is an expensive treatment, we must weigh the cost-effectiveness of SLED, given that we are a developing nation. For this reason, the purpose of this observational study was to ascertain the usefulness of SLED in our patient group. Our objective was to assess the efficacy of SLED in terms of the overall survival rate of the patients at a lower cost.

## Materials and methods

We enrolled 58 patients who were admitted to the critical care unit of the Pakistan Kidney and Liver Institute and Research Centre (PKLI-RC), Lahore, between January 1, 2023 and November 30, 2023, in this retrospective observational analysis. This 250-bed quaternary care hospital's closed ICU is overseen by an intensivist and contains 14 surgical, 13 medical, and four pediatric beds. All patients needing renal replacement treatment due to life-threatening diseases in critical care units were included, while patients less than 14 years of age were left out. Based on accepted indications (including acute kidney injury, acute on chronic renal failure, uremic encephalopathy, uremic pericarditis, hyperkalemia, refractory acidosis, volume overload, and hyperammonia in liver failure), hemodynamically unstable patients with mean arterial pressure (MAP) of < 65 mmHg and requiring vasopressors were offered sustained low-efficiency dialysis (SLED) as a form of renal replacement therapy. In addition, there were eight patients switched from intermittent hemodialysis (IHD) to SLED due to a drop in blood pressure (BP) after the commencement of IHD requiring vasopressor support. 

On average the duration of SLED lasted between six to eight hours per session daily. SLED treatments were discontinued at the discretion of the nephrologist if therapeutic objectives had been achieved by the time of the interruption. Most of our patients were pre-liver transplant, acute fulminant hepatic failure, renal failure, and septic with underlying coagulopathy, therefore SLED was done without anticoagulation and did not encounter any clotting issues. Dialysate (QD) and blood flow (QB) rates were set at 300 and 150 (range 100-200) mL/min, respectively. This makes it possible to gradually remove fluid and eliminate solutes by means of isolated ultrafiltration in diuretic-resistant volume overload patients when solute removal is no longer necessary. Our SLED protocol uses a potassium (3 mEq/L) dialysate bath which prevents significant hypokalemia. There was no significant hypophosphatemia observed in our patients which is usually an issue in CRRT post 24 hours of therapy when replenishment of electrolytes is needed. As acidosis in these patients is multifactorial, including lactic acidosis and or uremic acidosis, therefore blood flows and dialysis flows were increased as required so as vasopressors according to the hemodynamics of patients.

Documentation included clinical parameters at the time of admission to the ICU, standard laboratory testing, and demographic information on each patient (age and sex). Ultrafiltration volumes, patient vital signs, inotropic agent requirements, ventilatory support, and SLED prescriptions were all included in the therapy data. When a patient was admitted to the critical care unit, their level of illness was evaluated using the Acute Physiologic Assessment and Chronic Health Evaluation (APACHE) II score. The problems that the staff observed during SLED were also looked over and noted. Besides SLED all supportive treatment options were used as per need and to evaluate the prognosis. The primary objective was to measure the effectiveness of SLED and mortality during an ICU stay.

Categorical variables are expressed using percentages and frequencies, whereas continuous variables are expressed using the mean and standard deviation (SD). As a cutoff point, we classified the variables using the median value. The student t-test was employed for quantitative variables, and a p-value of less than 0.05 was considered statistically significant. The analysis was conducted using the software Statistical Package for Social Sciences (SPSS), version 20.0 (IBM Corp., Armonk, NY). The study was approved by the PKLI hospital's ethics committee (PKLI-IRB/AP/139).

## Results

The baseline features of our patients, along with relevant investigations, are displayed in Table 1. The mean age of 58 patients who had SLED was 48.6 ± 15.2. Of these, 44 (76%) were men and 14 (24%) were women. Among the patients, 30 individuals (51.7%) had chronic liver disease, 18 (31.1%) had chronic kidney disease, six (10.34%) had both chronic renal and liver disease, 19 (32.8%) had diabetes mellitus, 20 (34.5%) had hypertension, and two (3.5%) had ischemic heart disease. Among the patients, 32 had hepatic encephalopathy while 17 had hepato-renal syndrome (HRS) out of whom 12 passed away. Of the patients, six had both chronic kidney disease and chronic liver disease and 50 out of 58 patients (86.2%) had septic shock. A total of 117 SLED sessions were completed, with a median score of 2 (range: 1 to 7; mean: 2.02 ± 1.5).

**Table 1:** Characteristics of patients treated with sustained low-efﬁciency dialysis (SLED). ^1^ x̄ ± SD; all such values. ^2^ n = number of participant (%); all such values. ALF = Acute liver failure; CLD = Chronic liver disease; INR: International normalized ratio; HRS = Hepato-renal syndrome; CKD = Chronic kidney disease. APACHE II score = Acute Physiologic Assessment and Chronic Health Evaluation (APACHE) II score.

Variables (Unit)	Values
Mean age (years)	48.6 ± 15.2^1^
Males	44 (75.9%)^2^
Hemoglobin (g/L)	9.4 ± 2.4
WBC x 109/L	13.9 ± 8.6
Platelets x 109/L	179.8 ± 111.7
Sodium (mEq/L)	135.2 ± 6.2
Potassium (mEq/L)	4.9 ± 1.2
Bicarbonate (mmol/L)	15.8 ± 6.9
Creatinine (mg/dl)	4.7 ± 3.6
Lactate (mmol/L)	6.8 ± 5.6
INR (units)	2.15 ± 1.03
Ammonia (µg/dl)	141.65 ± 210.29
Albumin (g/dl)	2.38 ± 0.69
ALF	6 (10.3%)
CLD	30 (51.7%)
HRS	17 (29.3%)
Hepatic encephalopathy	32 (55.2%)
Grade 1	4 (6.9%)
Grade 2	2 (3.4%)
Grade 3	7 (12.1%)
Grade 4	19 (32.8%)
CKD	18 (31%)
CLD + CKD	6 (10.34%)
Mechanical ventilation	53 (91.4%)
APACHE II Score	28.8 ± 8.1
Septic shock	50 (86.2%)
Mortality	33 (56.9%)

Of the 58 patients who needed SLED (Table 2), the majority (86.2%) were in septic shock requiring vasopressors, refractory metabolic acidosis, and one or more organ failures (AKI = 32; ACLF = 11; ALF = 4; ACRF = 3). The remaining eight individuals experienced hypotension (MAP < 65 mmHg) while receiving intermittent hemodialysis and had to switch to SLED with vasopressors.

**Table 2:** Indications for SLED. ^1^ n = number of participant (%); all such values. AKI = Acute kidney injury; ACLF = Acute on chronic liver failure; ACRF = Acute on chronic renal failure; ALF = Acute liver failure; SLED: Sustained low-efficiency dialysis.

Indications	Number (%)	Mortality, n (%)
AKI	32^1^ (55.2%)	19 (59.4%)
ACLF	14 (24.1%)	8 (57.1%)
ALF	6 (10.3%)	3 (50%)
ACRF	6 (10.3%)	3 (50%)
Total	58	33 (56.9%)

As shown in Table 3 several modifiable factors that are independently associated with in-hospital mortality in critical patients receiving renal replacement therapy (RRT) were examined. A higher APACHE II score was associated with a greater mortality rate (p-value of 0.02) with a mean difference in APACHE II scores of 5.25 (95% confidence interval {0.87,0.96}). In addition, we found high international normalized ratio (INR) and low platelets are linked to increased mortality (p-values of 0.012 and 0.006, respectively), which is likely because most of them were not only with multi-organ failure but also had septic shock-causing coagulopathy. Unsurprisingly, septic shock carries a high mortality rate too, and the overall observed mortality rate was 56.9%.

**Table 3:** Prognostic markers. ^1^ x̄ ± SD; all such values. ^2 ^n = number of participant (%); all such values. INR: International normalized ratio. APACHE II score = Acute Physiologic Assessment and Chronic Health Evaluation (APACHE) II score.

Variables	Value	p-value
Mean age (years)		
Alive	48.2 ± 16.1^1^	0.87
Expired	48.9 ± 14.7	
APACHE II		
Alive	25.8 ± 9.3	0.02
Expired	31.1 ± 6.3	
Hemoglobin, g/L		
Alive	9.36 ± 2.25	0.94
Expired	9.42 ± 2.72	
WBC, x 109/L		
Alive	13.72 ± 9.23	0.91
Expired	13.97 ± 8.20	
Platelets, x 109/L		
Alive	226.4 ± 112.5	0.006
Expired	144.5 ± 98.7	
Sodium, mEq/L		
Alive	135.6 ± 4.03	0.68
Expired	134.9 ± 7.6	
Creatinine, mg/dl		
Alive	5.5 ± 3.98	0.14
Expired	4.02 ± 3.2	
Bicarbonate, mmol/L		
Alive	16.7 ± 7.8	0.4
Expired	15.1 ± 6.1	
Ammonia, µg/dl		
Alive	180.6 ± 261.8	0.299
Expired	116.7 ± 173.9	
Albumin, g/dl		
Alive	2.5 ± 0.7	0.369
Expired	2.3 ± 0.7	
INR, units		
Alive	1.8 ± 0.9	0.012
Expired	2.4 ± 1.1	
Lactate, mg/dl		
Alive	5.2 ± 5.7	0.072
Expired	8.2 ± 5.3	
pH		
Alive	6.5 ± 2.6	0.19
Expired	7.2 ± 0.16	
Septic shock (50)^ 2^		
Alive	17	0.001
Expired	33	

## Discussion

In ICU about 5% to 10% of AKI patients require CRRT [8,9], which has proven to be an effective treatment. However, in the last two decades, the number of patients requiring RRT for AKI has increased by approximately 10% per year [10], with mortality still remaining as high as 30% to 70% [11-15].

Sustained low-efficiency dialysis (SLED) is a hybrid form of CRRT and intermittent hemodialysis (IHD) with similar clinical outcomes [16]. The session lasts between eight and 16 hours in duration, with slower rates of solute clearance and ultrafiltration than IHD but faster than CRRT [17]. Generally, SLED equipment is the same as used for IHD, although it has lower flow rates for dialysate (350 mL/min) and blood (200 mL/min). The cost of the CRRT program is related to specialized machinery, filters and lines, and filtrate replacement fluid. SLED is at least 10-15% cheaper than CRRT in our setting as per treatment of SLED costs PKR 8500 (equivalent to USD $30) while CRRT costs PKR 150,000 (USD $535) on the first day followed by PKR 85,000/day (USD $330) till new tubing and filter are required, that on average last three days. This means three days of SLED treatment cost $90 vs CCRT cost of $1,195 at our center.

The indications for CCRT and SLED are the same as both are forms of RRT and there was no discernible difference in renal recovery. Although CRRT helps manage hemodynamically unstable patients, research to date does not show that CRRT, when compared to other treatments such as routine IHD and SLED, improves survival or restores kidney function [18]. SLED has comparable results to CRRT plus patients have more liberation if they need to be prone in case of acute respiratory distress syndrome (ARDS) or to transfers for CT or MR scan and physiotherapy. Most of our patients who were hemodynamically unstable tolerated SLED well, achieving modest solute clearances and ultrafiltration goals. However, some patients did require a brief increase in inotropic support that proved to be ephemeral, and hypotension that did not necessitate halting SLED could be readily managed. Between the CRRT and SLED therapy groups, there was no discernible difference in renal recovery, days to renal recovery, or hemodynamic instability, according to a meta-analysis [19]. These findings suggest that both approaches are safe and useful for treating critically ill AKI patients. On the other hand, a meta-analysis showed a 21% higher in-ICU death rate in critically ill patients with AKI receiving CRRT as compared to those receiving intermittent hemodialysis [20].

A study on 37 severely ill patients who failed IHD due to hemodynamic instability received 145 SLED sessions out of which 51 SLED treatments had to be interrupted early due to systemic clotting or hypotension, with an average duration of 10.4 hours; hospital mortality was reported at 62.2% [5]. Another study reported a survival rate of 37.5% only [21]. These figures match our 56.9% in-hospital mortality rate and the anticipated rates of in-patient death are in line with historical data on patients receiving RRTs for multi-organ failure [5,22,23]. Being primarily a transplant facility, it's also evident that most of our patients had underlying chronic kidney and liver diseases, which adds to the baseline guarded prognosis.

The in-hospital mortality rate for critically sick patients receiving renal replacement treatment is influenced by multiple risk factors including advanced age, lower body mass index (BMI), sepsis, the severity of the illness and the number of failing organs, hypernatremia, higher APACHE II and SOFA scores, the existence of circulatory shock, the need for mechanical ventilation and oliguria [15,24,25]. In contrast, we could not find any significant prognostic factor except a higher APACHE II score, high INR, and thrombocytopenia, which might be due to the modest number of participants in our study that may pose a barrier to its generalizability. Large-scale cohort studies will therefore be required in the future to identify the predictive factors in critically ill patients requiring renal replacement treatment.

Limitations

In this observational study, patient assignment to SLED was determined by cost-effectiveness and availability. Our sample is modest in number, and most of the patients already had underlying chronic liver or renal illness, which places them in a poor prognostic group. Even yet, our overall mortality rate is comparable to the historical data.

## Conclusions

In summary, considering our recent experience, sustained low-efficiency dialysis could be a viable option for selected patients who are hemodynamically unstable in whom intermittent hemodialysis had previously failed or been withheld. It is a cost-effective, safe, and convenient type of renal replacement therapy; however, a large-scale study is needed to further evaluate it in comparison with continuous renal replacement therapy.
